# *Mycoplasma genitalium* macrolide and fluoroquinolone resistance in pregnant women in Papua New Guinea

**DOI:** 10.1136/sextrans-2022-055552

**Published:** 2022-08-04

**Authors:** Marinjho E Jonduo, Andrew J Vallely, David M Whiley, Michaela A Riddell, William Pomat, Nicola Low, Emma L Sweeney

**Affiliations:** 1 Papua New Guinea Institute of Medical Research, Goroka, Papua New Guinea; 2 The Kirby Institute, University of New South Wales, Sydney, New South Wales, Australia; 3 Centre for Clinical Research, The University of Queensland Faculty of Medicine, Herston, Queensland, Australia; 4 Central Laboratory, Pathology Queensland, Herston, Queensland, Australia; 5 Institute of Social and Preventive Medicine, University of Bern, Bern, Switzerland

**Keywords:** mycoplasma genitalium, pregnancy, drug resistance, microbial, molecular diagnostic techniques

*Mycoplasma genitalium* is a sexually transmitted bacterium that colonises the human urogenital tract and in pregnant women has been associated with adverse pregnancy and birth outcomes.^1^ In pregnancy, the prevalence of *M. genitalium* is estimated to be 0.9% (95% CI 0.6% to 1.4%) in high-income settings and 12.5% in individual studies in low- and middle-income-countries.^1 2^ Globally, *M. genitalium* antimicrobial resistance (AMR) continues to escalate,^3^ and despite rising rates of AMR the prevalence of macrolide and fluoroquinolone resistance in *M. genitalium* has not been widely studied among pregnant women.^2^ Treatment of *M. genitalium* during pregnancy with selected antimicrobials such as macrolides is recommended, while tetracyclines are contraindicated and caution is recommended for fluoroquinolones,^4^ highlighting the potential issues surrounding available/effective antibiotics for this pathogen during pregnancy. In Papua New Guinea (PNG), macrolides and fluoroquinolones are routinely used for treatment of STIs^4^ and hence we aimed to characterise *M. genitalium* AMR in pregnant women.

We screened 69 *M*. *genitalium*-positive urine samples obtained from women attending their first antenatal clinic visit at five health facilities in Madang Province (PNG) between 2018 and 2019, enrolled in the control arm of a randomised controlled trial in PNG, the Women And Newborn Trial of Antenatal Interventions and Management.^5^ Confirmatory PCR was performed to verify the presence of *M. genitalium* in all 69 samples. Samples with positive results were then tested for the presence of AMR using targeted PCR assays. Macrolide and fluoroquinolone resistance/susceptibility markers were determined using previously published assays (online supplemental file). Stata V.15.1 was used to calculate the proportion of samples with resistance markers, with 95% exact binomial CI calculated using the Clopper-Pearson method. Where there were zero events, we give the 97.5% CI as the upper limit.

10.1136/sextrans-2022-055552.supp1 Supplementary data

From the five study sites, all 69 samples that were initially reported to be positive for *M. genitalium* were confirmed as positive by repeat PCR testing. Of these, 58 (84.1%) samples were successfully characterised for macrolide resistance, while 54 (78.3%) were successfully characterised for fluoroquinolone susceptibility/resistance (table 1). In summary, no samples with available results harboured macrolide resistance mutations (0.0%, 975% CI 0.0% to 6.2%) or the S83I fluoroquinolone resistance mutation (0.0%, 97.5% CI 0.0% to 6.6%), and all 54 samples were characterised as fluoroquinolone-susceptible (S83 wild-type).

**Table 1:** Results for macrolide and/or fluoroquinolone resistance in *Mycoplasma genitalium* isolates (N=69)

Study sites (location) (N=69)	Samples (n)	Average Ct value in *M. genitalium* PCR	SpeeDx ResistancePlus assay Macrolide resistance	TaqMan S83I and S83 assay Fluoroquinolone resistance		Summary
			23SRNA mutation	Wild-type*	S83I mutant	
Site 1 (rural), n=17	15	34.8	Not detected	Detected	Not detected	Characterised
	1	39.9	Not detected	No amplification	No amplification	Partially characterised
	1	38.5	No amplification	No amplification	No amplification	Not characterised
Site 2 (urban), n=11	6	35.5	Not detected	Detected	Not detected	Characterised
	1	37.9	Not detected	No amplification	No amplification	Partially characterised
	4	38.9	No amplification	No amplification	No amplification	Not characterised
Site 3 (rural), n=11	8	36.4	Not detected	Detected	Not detected	Characterised
	2	38.5	Not detected	No amplification	No amplification	Partially characterised
	1	38.5	No amplification	No amplification	No amplification	Not characterised
Site 4 (urban), n=11	8	36.8	Not detected	Detected	Not detected	Characterised
	1	39.2	Not detected	No amplification	No amplification	Partially characterised
	1	34.2	No amplification	Detected	Not detected	Partially characterised
	1	36.5	No amplification	No amplification	No amplification	Not characterised
Site 5 (rural), n=19	16	36.1	Not detected	Detected	Not detected	Characterised
	3	39.5	No amplification	No amplification	No amplification	Not characterised

Detected: sample was tested and we achieved an evaluable result (eg, no macrolide resistance observed and/or fluoroquinolone susceptibility was confirmed). No amplification: sample was tested; however, no evaluable result was obtained. Characterised: sample was successfully characterised by *both* assays. Not characterised: sample was *not evaluable by both* assays. Partially characterised: sample was successfully characterised by *one assay*.

*Wild-type (S83 sequence, no mutation) indicates fluoroquinolone susceptibility.

While data on *M. genitalium* AMR remain limited in many regions, including in low-income countries of the Western Pacific region, our findings indicate no evidence of macrolide or fluoroquinolone resistance in PNG. This further supports the findings of two previous studies of *M. genitalium* in PNG (78 pregnant women) and Solomon Islands (56 non-pregnant women) that also reported no macrolide resistance.^2 6^ Some of the reasons behind the lack of resistance in *M. genitalium* in these regions may include the fact that current STI treatment guidelines in PNG do not stipulate testing and treatment of *M. genitalium* in symptomatic persons.^4^ Other studies have identified that incorrect screening practices leading to treatment with macrolides have contributed to *M. genitalium*’s increasing resistance to macrolides.^7^ Given that both macrolides and fluoroquinolones are prescription drugs in PNG, their use in treating STIs and other infections is highly regulated. These drugs are not available for use in self-medication and so the repeated consumption/use of these drugs is not likely to be driving the selection of resistance in PNG, as has been suggested in other regions of the world.

From an STI management perspective, the clinical symptoms of *M. genitalium* infection are not distinct nor fully understood, as *M. genitalium* can be found in conjunction with other STIs, including gonorrhoea and chlamydia. In PNG, symptomatic patients often present with more than one curable STI. In this setting, the targeted testing and treatment for specific STIs is not routine and instead syndromic management of STIs is the standard of care. Resource-constrained health facilities throughout PNG use a single-dose cocktail of antimicrobials, including augmentin, amoxicillin, probenecid, tinidazole and azithromycin/erythromycin. An increase in presumptive treatment of these infections could result in the overuse of macrolides, which could indirectly lead to the development of AMR in *M. genitalium*. Additionally, asymptomatic women in PNG are less likely to attend sexual health clinics and therefore unlikely to receive either macrolides or fluoroquinolones, reducing the potential for *de novo* AMR.^8^

It is important to report on findings where AMR has not been detected to reduce the risk of overestimation of *M. genitalium* resistance in these settings. Future studies of *M. genitalium* AMR in PNG and similar settings are warranted, given the potential for import of resistant infections or for de novo resistance, if the use of macrolides and fluoroquinolones for reproductive tract infections increases.

## References

[1] Frenzer C , Egli-Gany D , Vallely LM , et al . Adverse pregnancy and perinatal outcomes associated with Mycoplasma genitalium: systematic review and meta-analysis. Sex Transm Infect 2022;98:222–7. 10.1136/sextrans-2021-055352 35351816 PMC9016252

[2] Scoullar MJ , Boeuf P , Peach E . High burden of Mycoplasma genitalium and other reproductive tract infections among pregnant women in Papua New Guinea. bioRxiv 2020. 10.3201/eid2703.201783 PMC7920647 33622474

[3] Machalek DA , Tao Y , Shilling H , et al . Prevalence of mutations associated with resistance to macrolides and fluoroquinolones in Mycoplasma genitalium: a systematic review and meta-analysis. Lancet Infect Dis 2020;20:1302–14. 10.1016/S1473-3099(20)30154-7 32622378

[4] Papua New Guinea National Department of Health and PNG Sexual Health Society . Papua New Guinea standard management of sexually transmitted infectionas and genital conditions. 2nd ed. Port Moresby, Papua New Guinea: Papua New Guinea National Department of Health, 2019.

[5] Vallely AJ , Pomat WS , Homer C , et al . Point-Of-Care testing and treatment of sexually transmitted infections to improve birth outcomes in high-burden, low-income settings: study protocol for a cluster randomized crossover trial (the wantaim trial, Papua New Guinea). Wellcome Open Res 2019;4:53. 10.12688/wellcomeopenres.15173.1 32030356 PMC6979472

[6] Harrison MA , Harding-Esch EM , Marks M , et al . Impact of mass drug administration of azithromycin for trachoma elimination on prevalence and azithromycin resistance of genital Mycoplasma genitalium infection. Sex Transm Infect 2019;95:522–8. 10.1136/sextrans-2018-053938 30981999 PMC6860407

[7] Kenyon C , Manoharan-Basil SS . Macrolide consumption and resistance in Mycoplasma genitalium. Lancet Infect Dis 2020;20:1235–6. 10.1016/S1473-3099(20)30727-1 33098774

[8] Vallely LM , Toliman P , Ryan C , et al . Prevalence and risk factors of Chlamydia trachomatis, Neisseria gonorrhoeae, Trichomonas vaginalis and other sexually transmissible infections among women attending antenatal clinics in three provinces in Papua New Guinea: a cross-sectional survey. Sex Health 2016;13:420–7. 10.1071/SH15227 28636866

