# Supplementary material for: Mycoplasma genitalium macrolide and fluoroquinolone resistance in pregnant women in Papua New Guinea
Source: Sex Transm Infect. 2022 Aug 4;99(1):71–2. doi: 10.1136/sextrans-2022-055552 (PMC9887348; doi:10.1136/sextrans-2022-055552)
Supplement: Supplementary data [file sextrans-2022-055552supp001.pdf]

Supporting information

Specimens

Stored midstream urine samples, collected from women attending their first antenatal clinic visit at five health facilities in Madang Province (PNG) between 2018 and 2019 were de-identified and stored at -80 °C and transported to the University of Queensland Centre for Clinical Research (UQCCR, Brisbane, Australia) for analysis. We identified 69 *M. genitalium* positive samples (unpublished data) from this cross-sectional study and further analysed them as part of this study.

Urine samples were thawed at room temperature and nucleic acid extracted using the Qiagen DSP virus/pathogen midi kit on the QIAsymphony SP/AS platform (Qiagen, Australia), according to manufacturers' instructions with the Complex400-V3 DSP protocol.

TaqMan Probe-based PCR assays

i) *MgPa* screening: *M. genitalium* primers and probe from a previous publication were used to detect *M. genitalium* within the clinical samples from this study.[1] In brief, reactions consisted of 12.5 µl Quantitect Probe mastermix (Qiagen), 10pmol of each primer, 0.2 µM probe, 5 µL of nucleic acid and PCR-grade water for a final volume of 25 µl. Samples were cycled using the Rotorgene 6000 (QIAGEN, Australia) real-time PCR instrument using: initial denaturation at 95°C for 15mins, followed by 50 cycles of 95°C for 15 sec and 60 °C for 60 sec.

ii) Fluoroquinolone resistance assay: *M. genitalium*-positive samples were screened for the presence of *parC* fluoroquinolone susceptibility and resistance markers using a previously developed assay.[2, 3] Reactions included 10 µl SensiFast Probe master mix, 0.5 µM of forward and reverse primers, 0.2 µM of S83 wildtype (FAM-labelled) and S83I (G248T; HEX-labelled) probes and 3 µL of nucleic acid extract in a total reaction volume of 20 µL. Each reaction included 5 µl nucleic acid extract and

Jonduo ME, *et al. Sex Transm Infect* 2023; 99:71–72. doi: 10.1136/sextrans-2022-055552

BMJ Publishing Group Limited (BMJ) disclaims all liability and responsibility arising from any reliance placed on this supplemental material which has been supplied by the author(s)

15µl of the prepared TaqMan mastermix and analysed using the Applied Biosystems 7500 Fast Dx Real-Time PCR instrument (Applied Biosystems®, Australia) using the following conditions: an initial hold at 95 °C for 5 minutes, followed by 50 cycles of 95 °C for 15s and 60 °C for 60s, acquiring data for both fluorescent probes on the annealing/ extension step.

SpeeDx Resistance*Plus*® MG PCR assay (Macrolide resistance)

A commercial qualitative real-time PCR assay was used to identify *M. genitalium* and detect the five most common mutations in the 23S rRNA gene (A2058G, A2059G, A2058T, A2058C, and A2059C, *Escherichia coli* numbering) that are associated with resistance to azithromycin (a macrolide antibiotic).[2,4] According to the manufacturer's instructions, each reaction constituted of 10 µl of Plex mastermix (2x), 1 µL of 23S mix, 1 µL control mix, 5 µL of nucleic acid extract in a total reaction volume of 20 µL. The reaction was analysed using the Applied Biosystems 7500 Fast Dx Real-Time PCR instrument (Applied Biosystems®, Australia) using the following conditions: initial denaturation of 95°C for 2min, followed by a 10 cycle touch-down cycling (initial denaturation at 95 °C for 5 seconds (sec), then 61°C – 56.5°C (-0.5 °C per cycle) for 30 sec) followed by 40 cycles of 95 °C for 5 sec and 52 °C for 40 sec (data acquired). The selected channels for data acquisition included: FAM (*MgPa* gene), JOE (23S rRNA mutation) and TAMRA (Extraction control).

Controls for PCR assays:

Well-characterised *M. genitalium*-positive clinical samples harbouring macrolide and fluoroquinolone resistance markers served as controls in all PCR assays, and nuclease free water in place of nucleic acid served as negative controls for all assays.

Reference

1. Jensen JS, Björnelius E, Dohn B, Lidbrink P. Use of TaqMan 5′ nuclease real-time PCR for quantitative detection of Mycoplasma genitalium DNA in males with and without urethritis who were attendees at a sexually transmitted disease clinic. J Clin Microbiol. 2004;42(2):683-92.

2. Sweeney EL, Lowry K, Bletchly C, Nimmo GR, Whiley DM. Mycoplasma genitalium infections can comprise a mixture of both fluoroquinolone-susceptible and fluoroquinolone-resistant strains. Journal of Antimicrobial Chemotherapy. 2021;76(4):887-92.

3. Tagg KA, Jeoffreys NJ, Couldwell DL, Donald JA, Gilbert GL. Fluoroquinolone and macrolide resistance-associated mutations in Mycoplasma genitalium. J Clin Microbiol. 2013;51(7):2245-9.

4. Su JP, Tan LY, Garland SM, Tabrizi SN, Mokany E, Walker S, et al. Evaluation of the SpeeDx ResistancePlus MG diagnostic test for mycoplasma genitalium on the applied biosystems 7500 fast quantitative PCR platform. J Clin Microbiol. 2018;56(1):e01245-17.

Jonduo ME*, et al. Sex Transm Infect* 2023; 99:71–72. doi: 10.1136/sextrans-2022-055552
